# Krüppel-Like Factor 4 (KLF4) Is Not Required for Retinal Cell Differentiation^1^,^2^,^3^

**DOI:** 10.1523/ENEURO.0117-15.2016

**Published:** 2016-02-27

**Authors:** Jiahua Fang, Peter X. Shaw, Yan Wang, Jeffrey L. Goldberg

**Affiliations:** 1 Department of Ophthalmology and Shiley Eye Institute, University of California San Diego, La Jolla, California 92093; 2 Department of Ophthalmology, Changsha First People’s Hospital, Changsha 410008, Hunan, China; 3 Byers Eye Institute at Stanford, Palo Alto, California 94303

**Keywords:** axon growth, Krüppel-like factor 4, retinal development, retinal ganglion cells

## Abstract

During early vertebrate eye development, a regulatory network of transcription factors regulates retinal cell differentiation and survival into adulthood. Among those factors, Krüppel-like factor 4 (KLF4) plays the dual role of maintaining the stem cell status of retinal progenitors cells and repressing the intrinsic axon regeneration ability in retinal ganglion cells (RGCs) after injury. This study further investigated whether KLF4 plays a role in early retinal cell differentiation or survival into adulthood. We examined different types of retinal neurons, including RGCs, amacrine cells, bipolar cells, Müller cells, and photoreceptor cells, in adult mice in which KLF4 was conditionally deleted in early retinal development using Chx10-promoted Cre by immunohistochemistry. We compared the numbers of retinal neurons and the thickness of photoreceptor and nerve fiber layers between Chx10–Cre-driven KLF4 deletion mice and wild-type mice. There was no significant difference in cell number among any of the retinal cell types or in photoreceptor layer thickness with KLF4 deletion during early development. The thickness of axon bundles in the nerve fiber layer in the Chx10 conditional KLF4 knock-out mice was greater than that in wild-type mice. These results suggest that KLF4 is not required for retinal cell differentiation or survival, but does normally limit retinal ganglion cell axon bundle thickness. These data support a hypothesis that KLF4 suppresses axon growth during development.

## Significance Statement

Although it has been well studied for its role in reprogramming somatic cells into induced pluripotent stem cells and the repression of axon regeneration of optic nerve injury, whether Krüppel-like factor 4 (KLF4) plays any role in retinal neuronal differentiation and survival into adulthood is still poorly understood. This study provides a line of evidence that the absence of KLF4 in early development does not affect retinal neuron differentiation into designated cell types.

## Introduction

The neural retina comprises six types of neurons (retinal ganglion cells, amacrine, horizontal, bipolar, and rod and cone photoreceptors) and one type of glial cell (Müller cell). They are generated from a common pool of multipotent retinal progenitor cells (RPCs). During vertebrate retina development, the differentiation of retinal cells occurs in a defined order (Cepko et al., 1996; Livesey and Cepko, 2001; Turner and Cepko, 1987). Retinal ganglion cells (RGCs) are generated first along with cone photoreceptors, amacrine cells, and horizontal cells, followed by bipolar neurons and rod photoreceptors, and ending with Müller glia, with considerable overlap among these phases (Cepko, 1999; Livesey and Cepko, 2001). In the early stages of retinal development, RPCs generate these different cell types under the regulation of a combination of extrinsic and intrinsic influences (Livesey and Cepko, 2001). The ability of RPCs to generate particular progeny is specified by transcription factors that define their competence to differentiate into single or perhaps subsets of retinal cell types (Cepko, 1999). Transcription factors known to regulate these functions include members of the basic helix-loop-helix (Hatakeyama and Kageyama, 2004; Yao et al., 2010), homeodomain (Dorval et al., 2005), and forkhead families (Moose et al., 2009).

Krüppel-like factors (KLFs) are a subfamily of evolutionarily conserved zinc finger DNA-binding transcription factors (Bieker, 2001; Ghaleb et al., 2005). Seventeen mammalian KLFs have been identified, all of which have three highly conserved zinc finger motifs at the C terminus; bind to similar consensus GT-box or CACCC DNA response elements; and function as transcriptional activators, repressors, or both (Dang et al., 2000; Moore et al., 2011; Turner and Crossley, 1999). These factors regulate various biological functions in development and adulthood, including cell growth, apoptosis, proliferation, and differentiation (McConnell and Yang, 2010). Different KLFs have been linked to the differentiation of different cell types, for example, KLF1 in erythropoiesis (Drissen et al., 2005), KLF2 in thymocytes (Carlson et al., 2006), KLF7 in olfactory sensory neurons (Kajimura et al., 2007), and KLF4 in monocytes (Alder et al., 2008) and corneal epithelial cells (Swamynathan et al., 2007).

KLF4 is expressed during murine eye development (Chiambaretta et al., 2004). When the KLF4 gene was selectively deleted in the surface ectoderm-derived structures of the eye with Pax6-driven Cre (le-Cre mice), 8-week-old mice exhibited multiple ocular defects, including corneal epithelial fragility, stromal edema, defective lens, and loss of conjunctival goblet cells (Swamynathan et al., 2007). We previously reported that KLF4 is developmentally regulated in RGCs and suppresses their intrinsic capacity for rapid axon growth (Moore et al., 2009). In that article, we did not see a defect in RGC numbers in adult mice after deleting KLF4 with a thy-1-promoted Cre in early development, but whether KLF4 regulates retinal neurons, including RGC differentiation, when deleted at the beginning of retinal development before Thy1 expression is turned on in postmitotic RGCs, remains to be determined. In this report, we created early retinal KLF4 conditional knock-out mice by breeding Chx10-Cre Tg with KLF4^fl/fl^ mice. Using immunohistochemical methods, we examined retinal architecture compared with age-matched wild-type (WT) controls.

## Materials and Methods

### Animals

All procedures involving animals were in accordance with the ARVO Statement for the Use of Animals in Ophthalmic and Vision Research and were approved by the Institutional Biosafety Committee and the Institutional Animal Care and Use Committee at the University of California, San Diego. Conditional knock-out mice were generated by breeding KLF4-floxed mice (Moore et al., 2009) and Chx10-Cre mice (Jax mice stock #005105, The Jackson Laboratory); genotyping was confirmed by PCR, as in our prior publication (Moore et al., 2009). Age-matched littermates without the Chx10-cre allele served as controls. For counting and statistical analysis of adult retinas, a minimum of three mice of either sex were included in each group.

### Western blot

Adult mice of indicated genotypes were killed, and retinas were dissected and lysed with lysis buffer (Cell Signaling Technology) containing 0.5 mm phenylmethylsulfonyl fluoride (Sigma-Aldrich). Protein concentration was determined by BCA protein assay (Thermo Fisher Scientific). Samples (25 μg) were separated by SDS-PAGE in 4-20% gradient Tris-glycine precast gels (Invitrogen) and transferred to a polyvinylidene difluoride membrane (Millipore). The membrane was incubated for 1 h in blocking solution containing 5% nonfat milk powder and 0.1% Tween-20, pH 7.6. This was followed by overnight incubation at 4°C in the blocking buffer containing rabbit primary antibodies against KLF4 (1:50; Ab72543, Abcam). Subsequently, the labeled proteins were visualized by incubation with a horseradish peroxidase (HRP)-conjugated anti-goat or rabbit IgG (1:2000; Santa Cruz Biotechnology) followed by development with a chemiluminescence substrate for HRP (Thermo Fisher Scientific). The images of Western blots were captured by GE ImageQuant. Relative band intensities were analyzed using ImageJ software and normalized to GAPDH.

### Immunohistochemistry

Animals under deep anesthesia underwent cardiac perfusion with 4% paraformaldehyde. Whole adult eyes were isolated, the corneas were incised to facilitate intraocular fixative penetration, and the eyeballs were transferred to PBS containing 4% paraformaldehyde for 1 h at room temperature, followed by 10% sucrose in PBS and, finally, 30% sucrose in PBS. Eyes were then mounted in optimal cutting temperature compound (Sakura Finetek USA), frozen, and cryosectioned at 5 μm thickness. At least three sections with an interval of 50 μm were acquired from each eye across the optic nerve to ensure retinal centration, for a total of 18 sections in each group. Cryosections were rinsed with PBS and blocked with 5% goat serum albumin (Thermo Fisher Scientific) and 0.2% Triton X-100 (Sigma-Aldrich) in antibody buffer for 1 h. Tissue was then incubated in antibody buffer containing primary antibody for 24 h at 4°C. The following antibodies were used: for RGCs, mouse anti-Brn3a (1:100; MAB 1585, EMD Millipore); for RGC axons, mouse monoclonal βIII-tubulin (1:100; Tuj1, Convance); for amacrine cells, mouse monoclonal anti-Pax6 (1:100; sc-81649, Santa Cruz Biotechnology); for photoreceptors and outer segment (OS) layer thickness, recoverin (1:100; EMD Millipore) and rhodopsin (1:100; Pierce/Thermo Scientific); for bipolar cells, rabbit polyclonal anti-protein kinase C-α (PKC-α; 1:100; sc-208, Santa Cruz Biotechnology); and for Müller glia rabbit anti-glutamine synthetase (GS; 1:100; Sigma-Aldrich). Sections were then rinsed in PBS and incubated with a fluorescent secondary antibody containing 4',6'-diamidino-2-phenylindole dihydrochloride (DAPI; 1:2000; Molecular Probes) for 24 h at 4°C for nuclei staining. The following two secondary antibodies were used in a dilution of 1:500: Alexa Fluor 488 mouse anti-rabbit Texas red or Alexa Fluor 546 goat anti-mouse (Molecular Probes). After a subsequent three washes in PBS, these sections were mounted with Vectashield H1000 (Vector Laboratories) and photographed with a Leica microscope.

### Morphologic analysis

Retinal cell counts were measured in cross sections to calculate cell density at a distance of 100-200 μm (both sides) from the center of the optic disc. The thickness of axon bundles in the ganglion cell layer (GCL) was quantified by measuring the thickness of the βIII-tubulin-immunostained RGC axons in the retinal nerve fiber layer at 200 μm from the center of the optic disc. Thickness of retinal layers containing photoreceptor cells plus outer segments (recoverin^+^ layer), and containing rod outer segments (rhodopsin^+^ layer) were also measured at 200 μm from the center of the optic disc. All measurements were collected by masked investigators.

### Statistical analysis

Data are presented as the mean ± SD with *N* = 18 replicates per condition; Student's *t* test (SPSS for Mac version 20.0) was used to test for significance at a *p* < 0.05 level.

## Results

### Early KLF4 deficiency does not affect photoreceptors into adulthood

**Figure 1.**
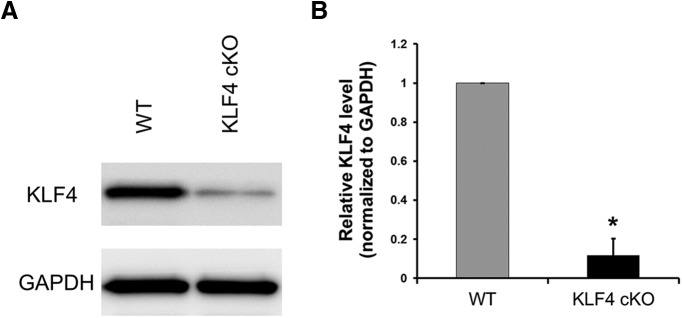
Western blot quantitative analysis of KLF4 protein expression in the retinae of KLF4 cKO and control mice. ***A***, Representative images of Western blots against KLF4 and GAPDH as marked. ***B***, Quantification of the relative protein expression normalized to total protein and GAPDH as marked (*N* = 3 for each biological sample; values are reported as the mean ± SD).

**Figure 2.**
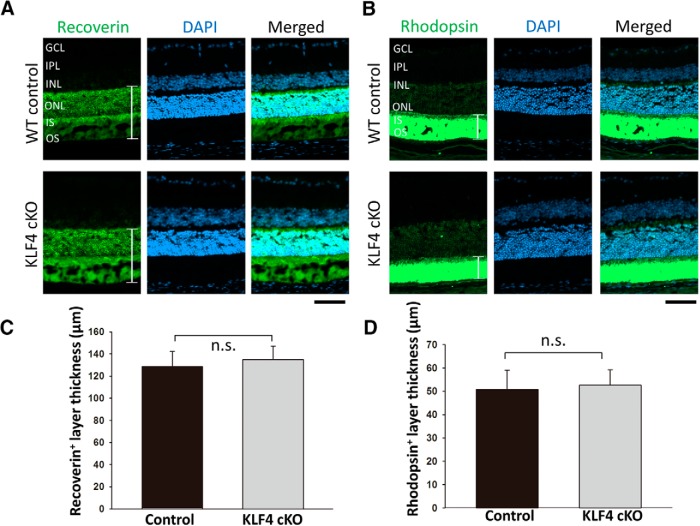
***A***, ***B***, KLF4 is not required for photoreceptors or outer segments. Immunofluorescence for recoverin (***A***) and rhodopsin (***B***; green) was counterstained with DAPI for nuclei (blue) in retina cross sections from wild-type control mice (top) and KLF4-cKO mice (bottom). IPL, Inner plexiform layer. ***C***, ***D***, The thickness of recoverin^+^ (***C***) and rhodopsin^+^ (***D***) layers (as marked with brackets in ***A*** and ***B***) were measured and found to be nonsignificant (n.s.; values are reported as the mean ± SD; *N* = 18). Scale bar, 50 μm.

We generated mice with retinas deficient in KLF4 by crossing a floxed KLF4 allele (Moore et al., 2009) with a cre recombinase-expressing line driven by the pan-retinal progenitor expressing marker CHX10 (Jax mice stock #005105, The Jackson Laboratory). Western blot analysis confirmed that the level of KLF4 protein expression in retinal tissues of KLF4^fl/fl^/Chx10-Cre^+^ mice was significantly decreased compared with that of the wild-type control mice (Fig. 1). To investigate whether the development of photoreceptors is influenced by retinal expression of KLF4, we examined recoverin on retinal cross sections of conditional knock-out (KLF4-cKO) and WT control mice. Recoverin is a calcium-binding protein that is present in both rod and cone photoreceptors (Polans et al., 1995). Both cKO and WT mice displayed intact photoreceptor cell somata, which showed very high levels of recoverin immunoreactivity throughout the outer nuclear layer (ONL), as well as normal-appearing inner segments (ISs) and OSs (Fig. 2*A*). We found no significant difference in the thickness of recoverin^+^ retinal immunostaining in WT versus KLF4-cKO mice (*p* > 0.1; Fig. 2*C*).

Rhodopsin is a single-chain integral membrane protein localized to rod outer segment membranes. Similar to recoverin, strong rhodopsin immunoreactivity was found in the photoreceptor outer segments of both WT and KLF4-cKO mice (Fig. 2*B*), with no significant difference in thickness between the two groups (Fig. 2*D*). Thus, both IS and OS photoreceptor measures are not dependent on retinal KLF4 expression.

### KLF4 is not associated with Müller, amacrine, and bipolar cell survival into adulthood

**Figure 3.**
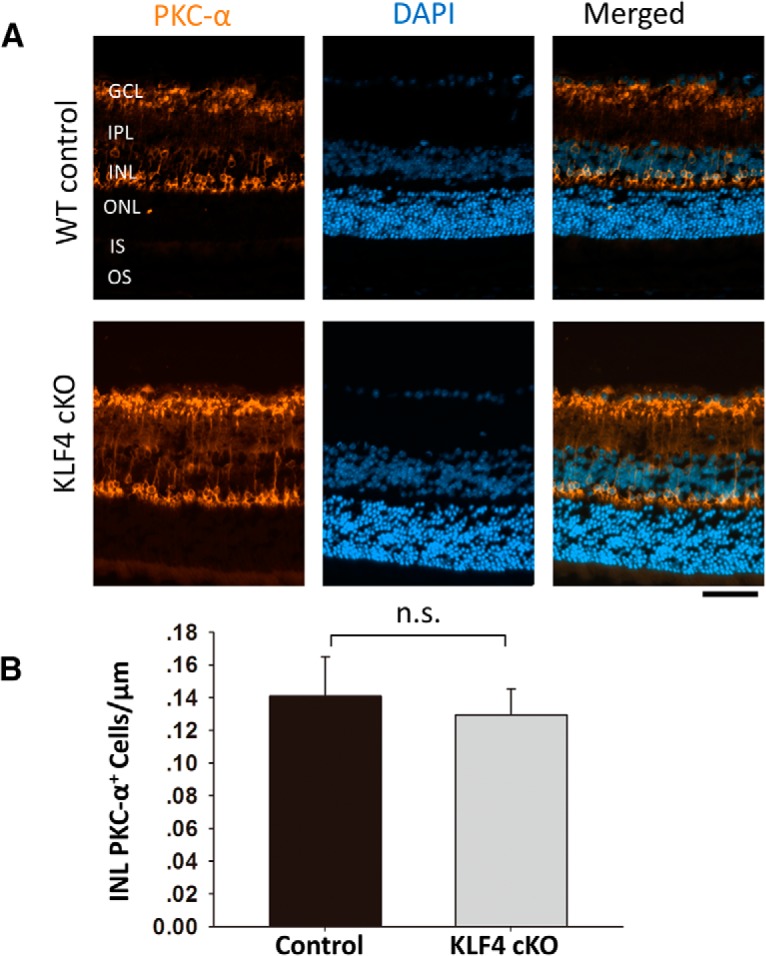
KLF4 does not affect rod bipolar cell numbers. ***A***, Retinal cross sections were immunostained for PKC-α (orange) and counterstained with DAPI (blue). ***B***, PKC-α^+^ cells in the INL showed no statistically significant difference between WT and KLF4-cKO groups (n.s.; values are reported as the mean ± SD; *N* = 18). Scale bar, 50 μm.

We next examined other retinal cell markers to determine whether early KLF4 deficiency affects differentiation and survival into adulthood. GS, an enzyme that converts neuron-released glutamate to glutamine, is a specific marker for Müller glia (Fischer and Reh, 2001; Jablonski et al., 2001). Immunostaining for GS demonstrated prominent labeling of Müller cell bodies located in the inner nuclear layer (INL) and their processes, extending to the inner and outer limiting membranes (Fig. 3*A*). GS^+^ cell bodies were counted only in the INL, not counting cells in the GCL wrapped by GS^+^ Müller glia end feet. We found no significant difference in Müller cell number between WT and KLF4-cKO mice (Fig. 3*B*; *p* > 0.1).

**Figure 4.**
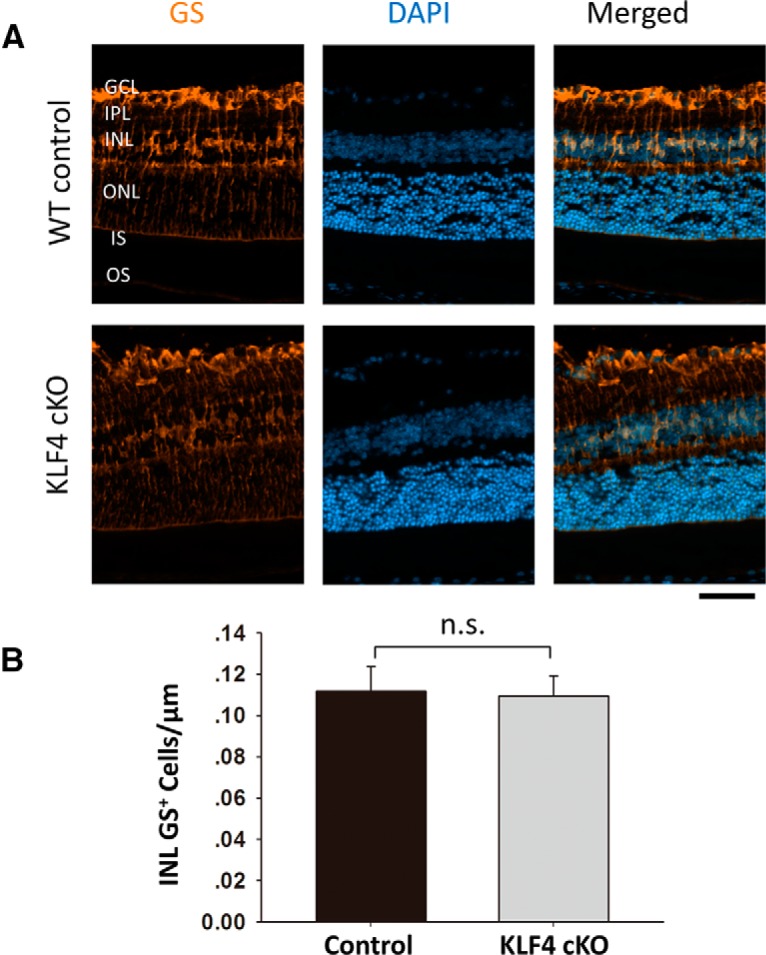
KLF4 does not affect Müller glial cell numbers. ***A***, Immunofluorescence staining was performed to detect GS (orange), and cells were counterstained with DAPI (blue). ***B***, The GS^+^ cells in the INL demonstrated no statistical difference between WT and KLF4-cKO groups (n.s.; values are reported as the mean ± SD; *N* = 18). Scale bar, 50 μm.

**Figure 5.**
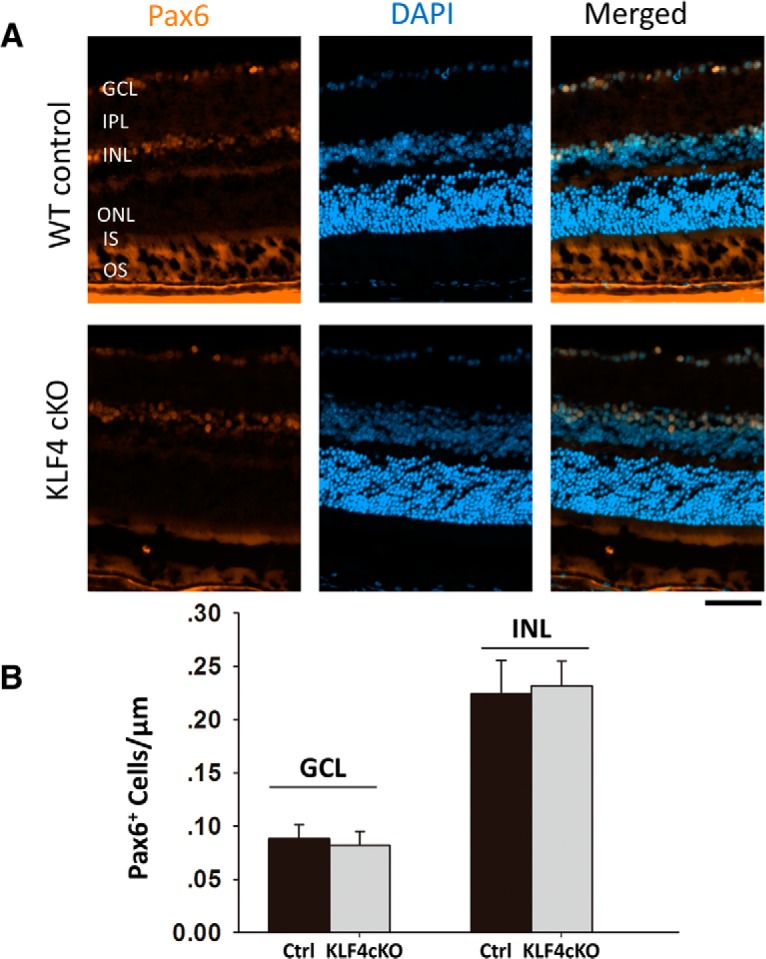
KLF4 is not required for amacrine cell numbers. ***A***, Immunofluorescence staining for Pax6 (orange) and counterstaining with DAPI (blue). ***B***, Pax6^+^ cells per unit length showed no statistical difference between WT and KLF4-cKO groups in either the GCL or INL (n.s.; values are reported as the mean ± SD; *N* = 18). Scale bar, 50 μm.

Using Pax6 as a marker for amacrine cells in the INL as well as the displaced amacrine cells in the ganglion cell layer (Fig. 4*A*), we found no significant difference between WT and KLF4-cKO mice (Fig. 4*B*). Using PKC-α, which is expressed in cell bodies and processes of ON bipolar cells that mainly connect to rods, we detected PKC-α immunoreactivity in a morphologically distinct population of rod bipolar cells with somata located in the distal row of the inner nuclear layer (Fig. 5*A*). Again, there was no difference in cell density of bipolar cells in WT versus KLF4-cKO mice (*p* > 0.05; Fig. 5*B*). Thus, KLF4 is not required to generate the normal number of bipolar, amacrine, or Müller glial cells in the adult retina.

### KLF4 is not associated with RGC differentiation and survival into adulthood

**Figure 6.**
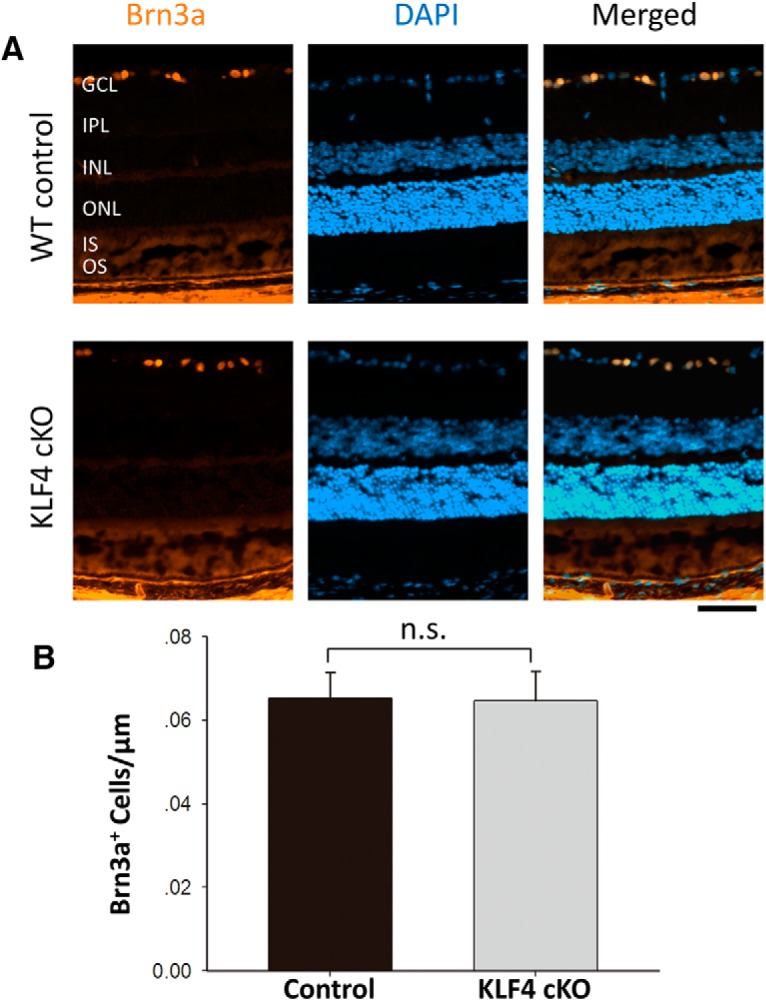
KLF4 is not required for RGC cell numbers. ***A***, Retinal cross sections were immunostained for Brn3a (orange) and counterstained with DAPI (blue). ***B***, Brn3a^+^ cells showed no statistically significant difference between WT and KLF4-cKO groups (n.s.; values are reported as the mean ± SD; *N* = 18). Scale bar, 50 μm.

We previously reported that, using a Thy1-Cre driver to disrupt KLF4 expression in approximately half of RGCs, RGC cell numbers into adulthood were not dependent on the postmitotic expression of KLF4 in RGCs themselves (Moore et al., 2009). Here, we extended those studies to determine whether KLF4 deficiency in progenitors of all retinal cells would affect RGC numbers into adulthood using the Chx10-cre allele. We used the RGC-specific marker Brn3a (Fig. 6*A*; Nadal-Nicolás et al., 2009) and found no significant difference in RGC numbers into adulthood (*p* > 0.05; Fig. 6*B*). Thus, for all retinal cell types studied, KLF4 deficiency in early retinal progenitor cells does not affect cell numbers or photoreceptor layer thickness into adulthood.

### KLF4 is associated with nerve fiber layer thickness

**Figure 7.**
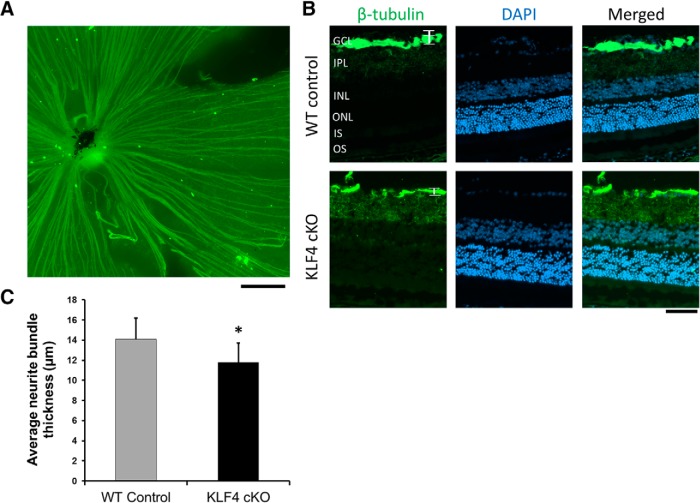
KLF4 deficiency leads to an increase in RGC axon fascicles in the adult retina. ***A***, Representative immunofluorescent staining of retinal flat mount. Scale bar, 200 μm. ***B***, Retinal cross sections were immunostained for β-tubulin (green) and counterstained with DAPI (blue). ***C***, The thickness of RGC axon fascicles (brackets in ***B***) was measured. There was a significant increase in nerve fiber layer axon bundle thickness in KLF4-cKO compared with WT mice (*p < 0.05; values are reported as the mean ± SD; *N* = 18). Scale bar, 50 μm.

Finally, because we and others previously found that KLF4 profoundly affects the ability of RGCs to regenerate their axons after optic nerve injury (Moore et al., 2009; Qin et al., 2013), we asked whether KLF4 affects RGC axon growth during normal retinal development *in vivo*. We used βIII-tubulin to stain RGC axons in flat-mounted retinas (Fig. 7*A*) and cross sections (Fig. 7*B*). Sections that traversed toward the optic nerve were analyzed. Due to the variability of nerve fiber bundles at the optic nerve head and the sparseness of axon bundles in the peripheral retina, we measured axon bundles 200 μm away from the optic nerve head to maintain consistency. By analyzing βIII-tubulin, which labeled fasciculated RGC axon bundles in the nerve fiber layer of the retina (Fig. 7*B*), we found that the thickness of axon bundles in KLF4-cKO mice is significantly increased compared to that of WT mice (*p* < 0.05; Fig. 7*C*). This result supports a model in which KLF4 expression normally suppresses some measure of axon growth *in vivo*.

## Discussion

During early vertebrate eye development, a regulatory network of transcription factors regulates retinal cell differentiation (Ohsawa and Kageyama, 2008) and survival into adulthood, including Pax6, Six3, Rx, Chx10, Notch, and Notch pathway components (Badea and Nathans, 2011; Mu and Klein, 2004). KLFs including KLF4 regulate differentiation of other cell types throughout the body (Apara and Goldberg, 2014; Moore et al., 2011), and KLF4 is one of a set of four factors capable of reprogramming somatic cells into induced pluripotent stem cells (Hanna et al., 2008; Takahashi et al., 2007; Takahashi and Yamanaka, 2006; Welstead et al., 2008; Yu et al., 2007). KLF4 plays an important role in cell cycle arrest and growth inhibition (Choi et al., 2006; Wei et al., 2005, 2008), and is highly expressed in the postmitotic cells of both the gut and skin (Shields et al., 1996; Zheng et al., 2009). KLF4 has been found to be downregulated in many types of cancers, indicating its possible contribution to cellular hyperproliferation and malignant transformation (Hu et al., 2009; Huang et al., 2005; Wei et al., 2005). However, in other types of primary tumors, such as breast cancer, the expression of KLF4 is upregulated (Foster et al., 2000; Pandya et al., 2004). An exact mechanism by which KLF4 affects negative or positive regulation in cell cycle control remains largely unknown. Progesterone receptor agonists can induce KLF4 expression and inhibit cyclin D1 expression, resulting in the inhibition of cell proliferation in human endometrial epithelial cells (Shimizu et al., 2010). Downregulation of cyclin D1 also controls the proliferation of retinal progenitor cells (Njaine et al., 2010). In addition, p21Cip1 coexpression may determine the tumor suppressor or oncogenic function of KLF4 (Rowland et al., 2005; Rowland and Peeper, 2006). The promoter region of KLF4 contains typical CpG islands, and, thus, hypermethylation may regulate KLF4 transcription and function in cell cycle control (Song et al., 2013).

In order to characterize the potential roles of KLF4 in retinal neuron differentiation and RGC development, we generated KLF4 conditional knock-out mice driven by Chx10-promoted Cre expression. We performed a Western blot analysis with retinal tissues from cKO and control mice. The results demonstrated the significant reduction (>80%) of KLF4 protein in Chx10 Cre-driven KLF4 cKO. The “escape” of a small amount of KLF4 expression in KLF4 cKO retinae may represent either Chx10-derived retinal cells not adequately excising the gene, or cells not derived from retinal progenitors, such as endothelial cells or nerve fiber layer astrocytes; this is a potential limitation of the cKO strategy. Because we did not find any differences in retinal cell numbers in the KLF4-deficient mouse retina, we hypothesize that KLF4 is dispensable for retinal progenitor cell differentiation and/or survival into adulthood. However, it remains plausible that KLF4 plays a role in both differentiation and survival into adulthood, and that alterations in both of these balance out to normal adult retinal cell numbers. The examination of earlier time points in retinal differentiation, for example at late embryonic and early postnatal ages, could assess this question in future experiments. It is also possible that KLF4 contributes to these processes but that other KLFs compensate in the absence of KLF4 (Moore et al., 2011). Furthermore, although we did not detect a difference in total Pax6^+^ amacrine cell numbers in the INL, amacrine cell subtype-specific immuno-identification (Kunzevitzky et al., 2013) could reveal changes in the differentiation of specific subpopulations.

RGCs and other CNS neurons in early development demonstrate robust axon growth and regenerative abilities, but their intrinsic capacity for rapid axon growth declines substantially after birth (Goldberg and Barres, 2000; Goldberg et al., 2002). We have recently reported that KLF4 plays a role in regulating intrinsic axon growth ability in RGCs (Moore et al., 2009). Overexpression of KLF4 significantly decreased neurite outgrowth in RGCs, hippocampal and cortical neurons, and RGCs lacking KLF4 showed increased axon growth both *in vitro* and after optic nerve injury *in vivo*.

Despite not affecting RGC numbers in the adult animal, in this study, we found that the thickness of RGC axon bundles adjacent to the optic nerve is increased in KLF4-cKO mice compared with WT adult mice. We declined to use optical coherence tomography (OCT), a promising technology particularly for longitudinal assessments of retinal layer thickness, as our data revealed subtle changes and OCT does not provide the resolution this available with histology and immunostaining. βIII-tubulin is a microtubule element expressed exclusively in neurons, and is frequently used to characterize RGCs and their axons in the retina. Other proteins such as neurofilament (NF) can mark RGC axons, but NF staining can vary between the axon hillock and the whole axon, depending on phosphorylation and the relative expression of NF-heavy, NF-medium, and NF-light chains, which vary in expression in different stages of development and after injury. Studying NF and other axon and synapse proteins would be an interesting area for future research in trying to determine how KLF4 suppresses axon growth *in vitro* and *in vivo*.

These data do not differentiate between axon number and axon caliber; an increase in either could explain these findings. Future experiments examining this question at the level of electron microscopy could differentiate between these possibilities, as well as to explore whether there are differences in dendrite elaboration *in vivo*, for example using single-cell imaging techniques. It would be interesting as well to explore whether these differences in nerve fiber layer thickness detected histologically could be detected in the adult mouse *in vivo* using noninvasive means such as OCT. Nevertheless, the finding of KLF4 function in suppressing some measure of axon development *in vivo* is consistent with our and others’ previous reports (Moore et al., 2009; Qin et al., 2013) that KLF4 normally acts to inhibit the intrinsic properties of neurite growth.

## Synthesis

The decision was a result of the Reviewing Editor Elizabeth Powell and the peer reviewers coming together and discussing their recommendations until a consensus was reached. A fact-based synthesis statement explaining their decision and outlining what is needed to prepare a revision is listed below. The following reviewers agreed to reveal their identity: Budd Tucker

The submission describes the role of KLF4 in early retinal cell differentiation and survival. While KLF4 is critical for stem cell growth, the role in retinal cell differentiation is yet unknown. The authors new data extend the field of knowledge and advances the field of retinal development and the overall roles of KLF4. The reviewers are enthusiastic about the manuscript, but they have a few issues that should be addressed. The individual comments are included:

Reviewer 1

In the manuscript titled "Kru¨ppel-like factor 4 (KLF4) is not required for retinal cell differentiation" the authors have investigated the effects of KLF4 in regulating retinal cell differentiation and survival. Their findings imply that KLF4 is not required for retinal progenitor cell differentiation and survival of retinal cells into adulthood. To identify the effects of KLF4, they have utilized CHX10-Cre driven KLF4 conditional knockout (cKO) mice and studied cell counts of retinal neurons: RGCs, amacrine, bipolar, Muller and photoreceptor cells. In addition, the authors have quantified the NFL/RGC axon bundle thickness.

Major revisions:

1.The authors suggest that KLF4 is not required for differentiation of retinal cells and cKO of KLF4 shows no significant difference in retinal cell counts between WT mice and the CHX10-Cre driven KLF4 cKO mice. However, the authors have only shown fluorescent microscopy images (Recoverin, Rhodopsin, GS, PAX6, PKC-a and Brn3a) 200um from the center of the optic disc. Please indicated if this analysis was perfromed in a masked fashion and why only 200um from the center of the optic disc was chosen?

Furthermore a discussion of how the different retinal layers were quantified and why the authors not quantify the size of the retinal fiber layers in vivo using OCT should be provided.

2.The authors should address in detail, the role of KLF4 in cell cycle control, and how it affects negative or positive regulation (Wei et al. 2008, Hu et al. 2009, Shimizu et al. 2010, Chen et al. 2010) and the effect in retinal progenitors (Njaine et al. 2010).

3.To quantify the thickness of the axon bundles, the only staining utilized was for BIII-tubulin. Would a neurofilament staining be better (For e.g. NFL)? The authors should also show cross-sections and traverse sections of the optic nerve to present a better case for axon bundle thickness.

For Figure 6, the authors mention, within the results (page 10) and discussion (page 11) sections that the axon bundles are significantly increased in the KLF4 cKO mice. However, the representative bracket of the GCL in figure 6 is thicker in WT than the KLF4 cKO. Please address.

Minor Comment:

There are several instances where the figures and figure legends throughout the manuscript do not match the results sections, please scan thoroughly and address as needed.

The authors are using PAX6 as a marker for amacrine cells, markers such as HPC1 and Calbindin may be useful.

Reviewer 2

1. I Would like to see confirmation that there is no expression of KLF4 in these animals by rq-PCR of total retinal RNA.

2. In figure 6, the B-tubulin stained bundles of k/o's are actually thinner than those of the WT control, although the graph shows the reverse, and the authors state that the k/o's have thicker bundles. Please replace photo with more representative confocal photo.

3. The authors suggest there may be some differences in mean axon diameter between WT and k/o animals. They suggest analysis of ON cross-sections (by TEM) might show difference in mean axonal diameter, if there is a shift in the type of axons, as well as simply looking at optical coherence tomography. It would enhance the paper's significance greatly if either of these approaches is included in the current report, and can be easily done (particularly with OCT).
